# Continuous, risk-based, consultation peer review in out-of-hours general practice: a qualitative interview study of the benefits and limitations

**DOI:** 10.3399/BJGP.2021.0076

**Published:** 2021-08-10

**Authors:** Ian Bennett-Britton, Jonathan Banks, Andrew Carson-Stevens, Chris Salisbury

**Affiliations:** Centre for Academic Primary Care, Bristol Medical School, University of Bristol, Bristol.; National Institute for Health Research Applied Research Collaboration West, Bristol.; Neuadd Meirionnydd, Division of Population Medicine, School of Medicine, Cardiff University, Cardiff.; Centre for Academic Primary Care, Bristol Medical School, University of Bristol, Bristol.

**Keywords:** delivery of health care, feedback, general practice, peer review, patient safety, primary health care

## Abstract

**Background:**

Systems to detect and minimise unwarranted variation in clinician practice are crucial to ensure increasingly multidisciplinary healthcare workforces are supported to practise to their full potential. Such systems are limited in English general practice settings, with implications for the efficiency and safety of care.

**Aim:**

To evaluate the benefits and limitations of a continuous, risk-based, consultation peer-review system used for 10 years by an out-of-hours general practice service in Bristol, UK.

**Design and setting:**

A qualitative study in South West England.

**Method:**

Semi-structured interviews with intervention users (clinicians, peer reviewers, and clinical management), analysed by inductive thematic analysis and integrated into a programme theory.

**Results:**

Twenty clinicians were interviewed between September 2018 and January 2019. Interviewees indicated that the intervention supported clinician learning through improved peer feedback, highlighting learning needs and validating practice. It was compared favourably with existing structures of ensuring clinician competence, supporting standardisation of supervision, clinical governance, and learning culture. These benefits were potentially limited by intervention factors such as differential feedback quality between clinician groups, the efficiency of methods to identify learning needs, and limitations of assessments based on written clinical notes. Contextual factors such as clinician experience, motivation, and organisational learning culture influenced the perception of the intervention as a support or a stressor.

**Conclusion:**

The findings demonstrate the potential of continuous, risk-based, consultation peer review to support clinicians in an increasingly multidisciplinary general practice workforce to efficiently and safely practise to their full potential. The programme theory provides a theoretical basis to maximise the benefits and accommodate the potential limitations of this methodology

## INTRODUCTION

Unwarranted variation^1^ in clinical practice is an area of increasing interest owing to the costs and harms of too much or too little health care.^2^ Within primary care internationally there is evidence of significant variation in clinician practice^3^ and a substantial burden of preventable harm.^4^^,^^5^ However, determining the extent to which observed variation is unwarranted and potentially harmful to patients is challenging, and requires detailed assessment of the clinician–patient interaction.^6^

Systems to identify and minimise unwarranted variation in individual clinician practice are increasingly relevant in the context of trends towards more multidisciplinary clinical workforces.^7^ Such initiatives have been adopted in England — as in other countries — to seek efficiencies, by ensuring all staff are working *‘at the top of their license’*,^8^ maximising the use of each team member’s skills.

Effective and standardised systems to detect and minimise unwarranted variation in clinician practice are crucial to ensure clinicians can be deployed and supported to practise to their full potential, rather than beyond their competence. Such systems are limited in English general practice settings,^9^ with implications for the efficiency and safety of care.

**Figure 1.**
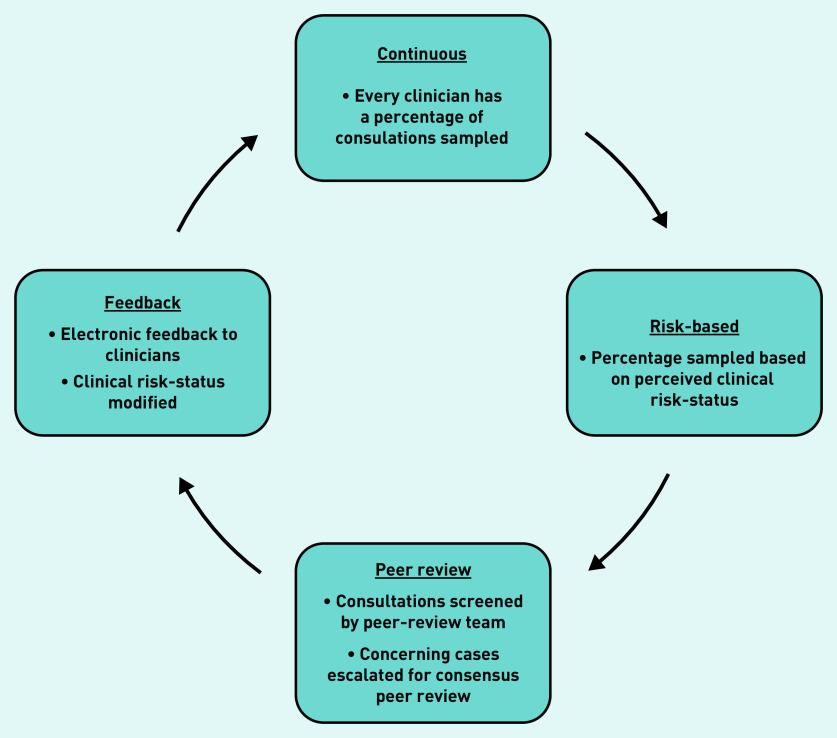
*The Clinical Guardian Intervention Cycle.*

A potential solution is a continuous, risk-based, consultation peer-review system developed and used by an out-of-hours general practice service provider in Bristol, England over the past 10 years. The ‘Clinical Guardian’ (CG) methodology^10^ (Figure 1) continuously samples a proportion of all clinicians’ consultation records for peer review. The proportion sampled varies between clinicians and is based on their clinical ‘risk-status’. ‘Risk-status’ is conceptualised as the degree of uncertainty regarding a clinician’s standard of practice, and is informed initially by time working with the organisation and subsequently by ongoing practice. Sampled consultation records are randomly selected and screened by members of a trained peer-review team with protected time to perform this function. Cases causing concern are escalated for detailed consensus peer review at regular team meetings. Case-grading, and where indicated constructive comments, are continuously fed back to clinicians through written electronic feedback to which they are encouraged to respond. Continuous modification of clinicians’ risk-status on the basis of their practice creates a feedback mechanism to focus the peer-review resource where it is most needed (see Supplementary Appendix S1 for details).

This study explored the benefits and limitations of CG to support the identification and minimisation of unwarranted variation in clinician practice in out-of-hours primary care, and informed the development of a programme theory to understand the impact of CG.

**Table:** How this fits in

Unwarranted variation in clinical practice is an area of increasing interest owing to the costs and harms of too much or too little health care. Effective systems to detect and minimise unwarranted variation in clinician practice are crucial to ensure clinicians in increasingly multidisciplinary healthcare workforces are supported to practise to their full potential. Such systems are limited in English general practice settings, with implications for the efficiency and safety of care. Continuous, risk-based, consultation peer review provides a mechanism to detect and minimise unwarranted variation in clinical practice, and a potential methodology to support clinicians to practise efficiently and safely.

## METHOD

### Setting

This study was undertaken in an out-of-hours general practice service in Bristol, England (BrisDoc Healthcare Services), and comprised interviews performed between September 2018 and January 2019. At that time the service served approximately 1 million patients and received >100 000 patient contacts annually.^11^ It was staffed by approximately 150 mostly self-employed GPs working flexibly, and 12 full-time equivalent non-GP clinical team members, alongside a clinical management team.^11^

### Methodological orientation

Semi-structured interviews were used to explore clinicians’ views on the benefits and limitations of CG. The intervention was perceived to be at the ‘development stage’ of the UK Medical Research Council guidance on the development and evaluation of complex interventions.^12^ This approach aimed to gather a breadth of perspectives to inform the generation of a programme theory,^13^ and to evidence whether the intervention merits further development.^14^

### Sampling

To triangulate viewpoints from those subject to the intervention, those who deliver it, and those who commission it, interviewees were purposively selected by role and experience across three groups:

- clinicians subject to the CG peer-review system (GPs, non-GP clinicians [NGP], and GP trainees [GPT]);
- CG peer-review team members (CGPRT); and
- senior management team (SMT) members involved in clinical governance.

### Recruitment

Interviewees were invited to participate via emails from their employer. GP trainees were invited to participate via emails from their training programme and a leaflet distributed at their teaching sessions.

### Data collection

Semi-structured interviews were undertaken by the lead researcher using interview topic guides (see Supplementary Appendix S2) exploring the benefits and limitations of the intervention. Specific questions about the role of CG in patient safety, care quality, clinician learning, and the identification of clinicians in need of support were asked to elicit views on areas hypothesised to be important a priori. Written or audiorecorded verbal consent was gained before each interview. Interviews were recorded using an encrypted digital audiorecording device and interviewees were offered a 40 GBP gift-voucher to thank them for their time.

### Data analysis

Interviews were transcribed, anonymised, and checked against each recording to ensure accuracy. Transcripts were analysed using inductive thematic analysis;^15^ however, the lead author’s knowledge of quality assurance structures in general practice^9^ may have engendered a level of deduction. Initial codes were generated through review of all transcripts. Codes were reviewed, grouped into themes, and checked to ensure congruence with individual coded extracts and the overall dataset. Two researchers independently coded a subset (15%) of the transcripts to ensure consensus over coding and generation of themes (see Supplementary Box S1 for details). Analysis was organised using NVivo (version 12) qualitative data analysis software.

Sampling of clinicians was based on an initial target of 18 interviews, informed by previous experience of qualitative research. No new themes were emerging on completion of this target except in relation to clinician experience, leading to two further interviews. The preliminary findings were presented to nine members of the CG senior management and peer-review team to support service development and as an opportunity for feedback. No objections to the preliminary findings were raised and no new themes emerged, supporting the authors’ assessment of data saturation.

**Figure 2.**
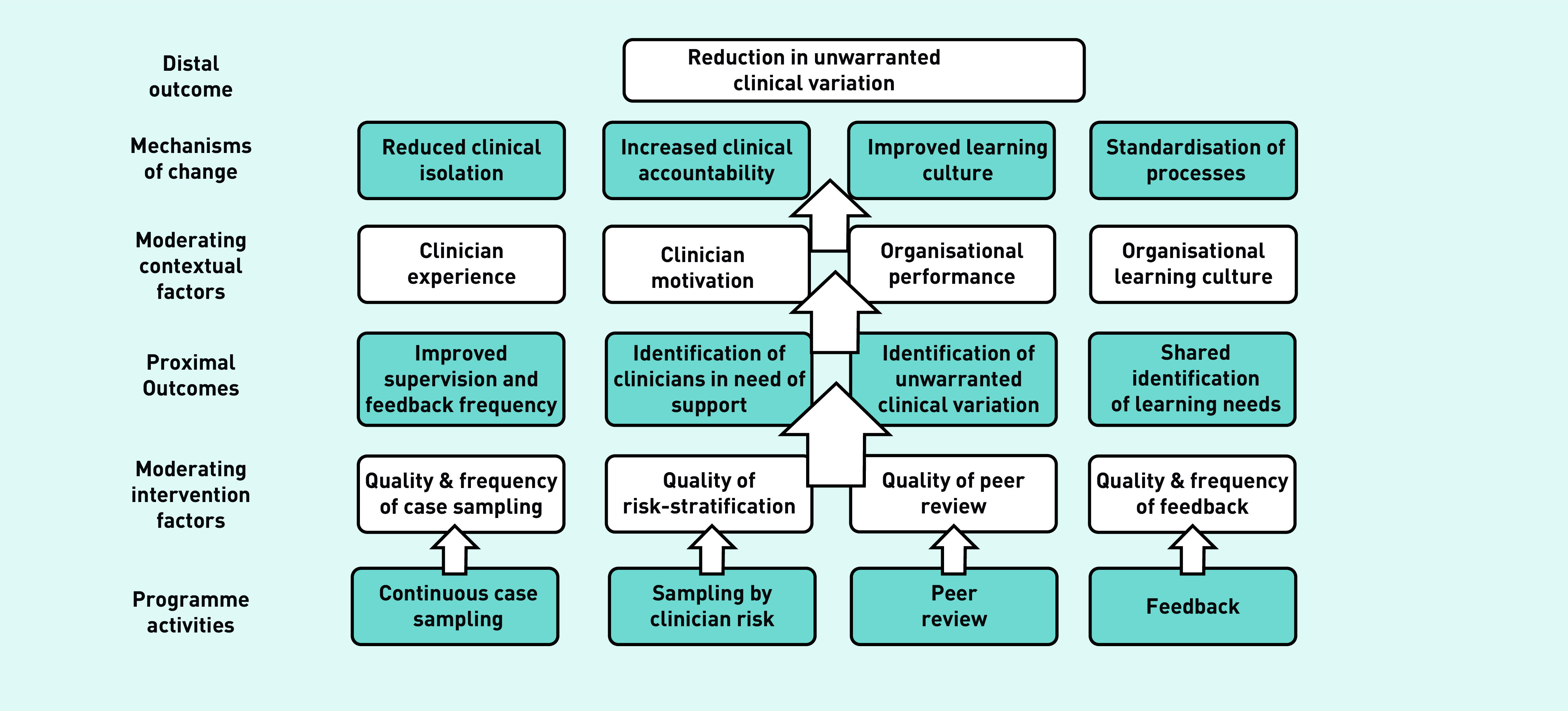
*Intervention programme theory.*

To ensure a theoretical basis for further enquiry, the findings were synthesised to produce a programme theory (Figure 2).^13^^,^^16^

## RESULTS

Twenty clinicians were interviewed, with interviews lasting 23–52 min. The distribution of participants’ roles are summarised in Table 1. Most interviewees (85%) worked in both in-hours and out-of-hours general practice at the time of interview, and nearly all had previous experience of in-hours general practice (90%). Interviews were undertaken face-toface at the University of Bristol (*n* = 3), the clinicians’ workplace (*n* = 3), or by telephone (*n* = 14) (data not shown).

**Table 1.** Interviewee distribution by role

**Interviewee role**	**Number of interviewees** **by CG role**	**Number of interviewees** **by clinical role**
**SMT**	3	

**CGPRT**	3	

**Clinicians subject to CG**		
GP	8	13
Non-GP clinicians	3	4
GP trainees	3	3

**Total**	20	20

*CG = Clinical Guardian. CGPRT = Clinical Guardian peer-review team. SMT = senior management team.*

### Benefits of the CG intervention

The benefits of CG pertained to themes of supporting clinician learning, ensuring clinician competence, and organisational quality assurance.

#### Supporting clinician learning

a) Peer feedback levels: many of those with experience of in-hours general practice noted peer feedback to be infrequent in that setting. By comparison, CG was felt to have a positive impact on feedback frequency, circumventing many identified causes of infrequent feedback such as time, clinical isolation, professional hierarchy, and avoidance of conflict:

‘ [CG is] *the only feedback I really get on my documentation … my colleagues are probably too nice, and they’re not in a rush to offer me that feedback.’*

(GP8)

b) Identification of learning needs: clinicians recognised inconsistencies in their own practice and how minimising this could improve care:

*‘… there’s a high rate of variation even in my own practice so I think anything we can do where we spot inconsistencies, could potentially improve quality.’*

(GP1)

In contrast with existing, predominantly reactive quality assurance structures, CG supported proactive identification of clinicians’ learning needs:

*‘At the moment, the way people’s unknown unknowns get picked up is some sort of significant event or complaint when something has gone wrong. Otherwise, it just passes under the radar … ’*

(GP7)

Owing to its frequency and detail, CG facilitated feedback regarding issues that would otherwise not be highlighted, with potential patient benefit through optimisation of existing clinical management:

*‘* [Without CG] *you don’t get the sort of small bits of feedback or slight nudges to improve like, “make sure you document your safety netting.” I mean who is ever going to spot that otherwise?’*

(GPT2)

c) Validation of practice: CG was identified as a means of validating clinician practice and benchmarking with peers *.* The use of CG to reflect on how clinicians’ actions correlated with patient outcomes provided a mechanism to reinforce positive practice or trigger learning *.*

#### Ensuring clinician competence.

a) Clinician supervision: CG was felt to ensure a minimum standard of supervision for a professionally diverse and often transient out-of-hours general practice workforce:

*‘…* [CG is] *really crucial because you’re employing a* [range] *of clinicians, and a lot of them are nurses and paramedics. So, how on Earth do you check somebody is alright? You have a responsibility to the patients to ensure … you’re checking up on standards … ’*

(CGPRT1)

Owing to infrequent peer feedback in in-hours general practice, many clinicians noted CG feedback on their out-of-hours role was the only form of clinical supervision they received. Many felt the accountability provided by such supervision was likely to improve clinical practice:

*‘… there could be an element of … if you know that someone is checking your work, you might be a bit more thorough. There shouldn’t be, but there probably is.’*

(GP7)

There were concerns that CG could be seen as a replacement for supervision that should be more formalised and detailed:

*‘I think the problem with doing Clinical Guardian is it could too easily become a substitute for something that should be a lot better.’*

(GP6)

Inadequate supervision was recognised to have greater consequences for those with less clinical experience, and in the context of an increasingly multidisciplinary general practice workforce, risked deploying clinicians outside of their competence.

Acknowledging those concerns, participants emphasised competence to be a function of clinical practice rather than something to be inferred by professional title, and recognised value in supervision structures that apply equally to all clinicians:

‘ *... it doesn’t actually matter whether it’s a GP or not because we are all doing the same job, so we all have to be competent … ’*

(GP6)

While CG may not provide a gold-standard of supervision for all clinicians, there was recognition that it represented an improvement on the perceived lack of consistent approaches in in-hours general practice:

*‘ …* [CG] *gives a … solidity to the service in terms of that there is a running check of records of every clinician. In-hours general practice is 55 miles from that.’*

(SMT2)

b) Identification of clinicians in need of support: while the number of performance outliers were noted to be low, CG was felt to be an effective mechanism to identify clinicians in need of support:

*‘If you get … someone who is really not doing the things they should do, it’s a really effective way of just picking that up.’*

(CGPRT1)

Continuous sampling of clinician practice was seen as a strength of CG, enabling the identification of patterns of behaviour that may highlight a need for support, and the universal application of CG ensured supervision of those who might not otherwise seek it.

c) Appraisal and revalidation: most clinicians supported the rationale for nationally standardised structures to ensure clinician competence, such as appraisal and revalidation. However, many did not feel such mechanisms were effective, and the reliance of appraisal on largely self-collated information was noted to create adverse incentives that may undermine it:

*‘… I am much less likely to pull out cases where to be honest I am pretty sure I haven’t done the best thing* [in my appraisal], *than I am … with my colleagues.’*

(GP6)

Clinicians indicated CG enhanced the quality of evidence they could submit for appraisal, and was potentially less biased than other means of assessing clinician competence, owing to its risk-based case sampling and equal application to all clinician groups.

#### Organisational quality assurance

a) Clinical governance: participants with experience of working for >1 service provider noted interorganisational variation in clinical governance culture and practices, with patient safety incident reporting identified as an area of inconsistency. CG was seen to help standardise such approaches.

Participants indicated clinical governance could be better integrated between organisations, and standardising approaches with the support of systems such as CG was seen as a way to facilitate interorganisational learning.

b) Organisational learning culture: for many, CG supported a positive learning culture and sense of organisational connectedness, which was noted to be harder to achieve in larger organisations. Some felt CG introduced a sense of hierarchy; however, this viewpoint was not widely held, and sharing learning from CG reviews at an organisational level via meetings and emails facilitated the perception of CG learning as a team exercise. Investment in CG was felt to communicate organisational values to clinicians, external organisations, and patients.

### Factors limiting the usefulness of CG

Factors limiting the usefulness of CG pertained to the intervention itself, the clinician, and organisational context.

#### Intervention factors

a) Feedback quality and frequency: feedback containing written comments, rather than categorical grading, was perceived as being most useful for clinician learning. CG was recognised to focus on those most in need of support; therefore, many did not receive detailed feedback regularly, attenuating its use to them as a learning tool.

Constructive feedback was recognised to be of greatest value in supporting learning, but a potential source of anxiety and defensiveness:

*‘It’s really easy to give positive feedback. It’s quite difficult to broach the thorny issue of trying to suggest somebody does things in a different way.’*

(CGPRT1)

These concerns were rationalised in terms of concerns about the opinion of peers; that their actions could have caused patient harm and the associated medicolegal implications.

b) Selection of clinical cases: CGs’ use of random consultation record sampling was felt to reduce bias; however, some highlighted learning-points may be more efficiently identified through more purposive case selection. Some noted that when they had self-selected challenging cases for peer review this was highly valued.

c) Limitations of clinical note reviews: the CG peer-review process is principally conducted using consultation records. Interviewees recognised these to be a subjective representation of a clinical interaction from the clinician perspective, and therefore a potential limitation:

*‘… notes aren’t the whole picture, it’s the way that the GP documents the kind of transaction … ’*

(GP5)

Consequently, clinical records were observed to be vulnerable to unintentional or intentional misrepresentation.

Overemphasis on the clinical note quality may neglect other important aspects of practice such as consulting speed, breadth of competence, and quality of communication. Notes may also not adequately reflect the context of a clinical encounter, where difficult decisions may be made on a busy shift:

*‘… there’s always going to be a limitation where there’s only one person at a distance looking at something you’ve done without the context or the business of the shift … ’*

(GPT1)

Despite these limitations, the strength of notes reviews to appraise the key points of a clinical interaction supported its role as a quality assurance tool:

*‘…* [CG is] *not really digging into what’s actually going on in the consultation … but yet it’s useful because you can see … that they’re taking good sets of records, taking appropriate clinical observations, and taking an appropriate course of action for a defined clinical problem.’*

(SMT1)

#### Clinician factors

a) Clinician experience: less experienced clinicians appeared to find CG more useful as a consequence of having a greater proportion of their consultations reviewed than more experienced colleagues and being trained through a similar culture:

*‘… younger GPs who’ve come through … training where they’re used to reflecting, being observed, getting lots of feedback … in general seem to find* [CG] *more useful ... ’*

(SMT3)

Some more experienced clinicians felt they should be audited less; however, most reflected they would value more feedback, and experience did not negate the need for scrutiny:

*‘There’s lots of GPs who maybe qualified 40, 45 years ago, don’t write very detailed notes. There has been a change in what is considered appropriate so hopefully* [CG] *encourages that.’*

(GP7)

b) Clinician motivation: Clinicians were noted to be broadly receptive to peer-review interventions owing to common professional traits:

*‘… doctors tend to get … very reflective … and very self-critical. They’re high achievers who’ve got quite high standards.’*

(CGPRT1)

Interviewees recognised the focus of CG was to support clinicians, rather than catch them out. Most reflected a preference for more peer review of their practice *,* with those most committed to self-development finding CG most useful.

#### Organisational factors

a) Organisational performance: the potential impact of CG was noted to be affected by the strength of wider governance structures and existing organisational performance.

b) Learning culture: while some felt monitoring their practice through CG was within the spectrum of assurance processes they would expect in any clinical service, others noted the potential impacts of such interventions on the health and retention of already stretched clinicians:

*‘... you have to balance, don’t you, patient safety and doctor morale ... being overly watched and scored is a big factor in doctor morale and burnout and stress ... ’*

(GP3)

These tensions highlighted the importance of learning culture in influencing perception of such interventions. While CG was reported to promote many positive aspects of organisational learning culture, it was emphasised as a tool to support this, rather than a substitute, and the critical role of organisational leadership in setting such a culture was recurrently noted:

*‘… clinical governance* [is] *… about culture and climate and permission to fail … it is the senior people who set that climate for better or worse. If* [CG] *is ever going to be taken further, that has to be a focus.’*

(SMT2)

## DISCUSSION

### Summary

This study evaluated the usefulness and limitations of risk-based, continuous, consultation peer review in identifying and minimising unwarranted variation in clinician practice. The findings have been incorporated into the programme theory presented in Figure 2.

Interviewees indicated the intervention supported clinician learning through improved peer feedback; highlighting learning needs and validating practice. It was compared favourably with existing structures of ensuring clinician competence; supporting standardisation of supervision, clinical governance, and learning culture.

These benefits were potentially limited by intervention factors such as differential feedback quality between clinician groups, the efficiency of methods to identify learning needs, and limitations of assessments based on written clinical notes. Contextual factors such as clinician experience, motivation, and organisational learning culture influenced the perception of the intervention as a support or a stressor.

### Strengths and limitations

This study interviewed clinicians involved in all aspects of this intervention that had been established for 10 years, representing an evaluation of embedded use. The structure, consistency, and longevity of CG provides a valuable example to aid others to consider adopting or adapting such approaches.

Employees may have felt pressure not to criticise the intervention; however, the authors sought to minimise this through reassurance of anonymisation. This study was undertaken in a single organisation; therefore, contextual factors such as organisational size and learning culture should be considered before generalising findings.

### Comparison with existing literature

Participants’ reports of limited and inconsistent quality assurance processes in in-hours general practice were in keeping with other studies,^9^^,^^17^^,^^18^ and set in the context of evidence of a substantial burden of preventable patient harm in English primary care.^5^ While this study has indicated the potential of CG to support an increasingly multidisciplinary primary care workforce to practise to its full potential, the findings also reflect the need to ensure such interventions do not become another stressor for a workforce dealing with a challenging and rising workload.^19^

Consultation peer-review interventions such as CG are a form of clinical audit. Estimates of the effects of audit and feedback on professional practice and patient outcomes in health care are challenged by the heterogeneity and quality of studies in this area.^20^^–^^24^ Despite this, characteristics associated with interventions that are most effective have been identified.^20^^,^^24^ While the scope of this study did not encompass the analysis of CG feedback necessary for comprehensive comparisons with such frameworks, CG is consistent with many characteristics identified as important, such as targeting of low baseline performance,^20^ and providing contemporaneous, individualised, regular feedback from a trusted source with whom one can socially interact through reply.^24^ These synergies suggest some of the active mechanisms through which CG may be effective, reflecting the components of the programme theory (Figure 2), and along with more comprehensive theories of healthcare feedback,^25^ providing a basis for future intervention development and evaluation.

The use of patient record review to measure and improve patient safety in general practice is longstanding,^26^^,^^27^ with most studies using a screening methodology to target the peer-review resource most efficiently at cases of interest. Studies using these methods to target consultations containing patient safety incidents in UK general practice settings have consistently found previously unidentified safety incidents and preventable harm.^28^^–^^32^ However, the impact of such interventions on patient outcomes is unclear.^30^^,^^31^

CG differs from most patient record review approaches in its use of clinician risk to inform case sampling. Doing so is supported by evidence indicating relatively small proportions of clinicians account for a disproportionate burden of negative outcomes, such as patient complaints.^33^ CG also differs from most peer-review interventions in its use of a trained peer-review team with regular protected time to perform this function, likely contributing to the unusual longevity and consistency of its use. Such an approach is supported by findings of previous studies that found uptake and consistency of peer-review interventions to be affected by time available, resources, peer-reviewer skills, and the extent of integration with other activities.^34^

Clinical audit involves the measurement of practice against professional standards or targets.^20^ The lack of explicit standards for numerous aspects of routine care, and breadth of reasons for appropriate deviation from such standards where they exist, highlight the value of a quality discourse that focuses on warranted and unwarranted variation, rather than measures of adherence to specific targets.

Sutherland and Levesque^1^ have proposed a helpful framework to understand and analyse warranted and unwarranted variation in health care in terms of the use of evidence, allowance for individual agency, and clinician and service capacity. Their framework integrates patient, clinician, organisational, and wider contextual factors, and emphasises the need to consider how variation is attributed and aggregated. CG enables such a nuanced approach through consensus peer review by multiple reviewers to discern whether actions taken by a clinician — such as choosing whether to admit a patient to hospital or prescribe a particular medication — are likely to be warranted. Such systems accommodate the complexity and uncertainty of decisions in health care.

In keeping with the proposed ‘mechanisms of change’ (Figure 2), other commentators have hypothesised that interventions that facilitate professional collaboration through feedback enable sharing of best practice, provide accountability through making ‘individual behaviour visible’, and reinforce organisational learning culture.^35^ Uncertainty regarding best practice has also been highlighted as a contributor to variation,^35^ and continuous peer-review interventions such as CG help highlight areas of uncertainty to be targeted by organisational guidelines and further research.

### Implications for research and practice

Effective and standardised systems to detect and minimise unwarranted variation in clinician practice are necessary to ensure clinicians in an increasingly multidisciplinary primary care workforce can be safely and efficiently deployed and supported to practise to their full potential. This study suggests that continuous, risk-based, consultation peer review provides a potential mechanism to achieve this aim.

Such systems have the potential to transform the regulation of the healthcare workforce by shifting from an emphasis on historical training and professional identity^9^ towards a focus on ensuring and developing clinician competence. This approach may provide a framework to reward skill development though alternative training routes or reduce the scope of practice of those in need of support.

Further research is needed to understand how CGs’ proposed ‘mechanisms of change’ (Figure 2) can be optimised. This will be achieved through development of an intervention to enable the identified ‘moderating intervention factors’ and a better understanding of how the form of the intervention may be flexibly adapted to accommodate the ‘moderating contextual factors’ of each target environment.

Exploring and integrating factors such as the patient voice, wider clinician performance data, and the role of such systems in promoting continuous learning at the clinician, organisation, and health system level may form the basis of the *‘more sophisticated conversations about audit and feedback to achieve substantial, data driven, continuous improvement’*,^23^ which are needed to revitalise this research area, minimise unwarranted variation, and improve the efficiency and safety of health care.
